# Bloom Syndrome Presenting as Early-Onset Bilateral Breast Cancer and Multisystem Metabolic Disease: A Case Report

**DOI:** 10.7759/cureus.112078

**Published:** 2026-07-05

**Authors:** Floor Bracquez, Corina E Andreescu, Laura Iconaru, Olga Kosmopoulou, Jeroen De Filette

**Affiliations:** 1 Diabetes and Endocrinology, Universitair Ziekenhuis Brussel (UZ Brussel), Brussels, BEL; 2 Diabetes and Endocrinology, Centre Hospitalier Universitaire Brugmann (CHU Brugmann), Brussels, BEL

**Keywords:** blm gene, bloom syndrome, breast cancer, consanguinity, lipodystrophy, multigene testing, rare disease

## Abstract

Bloom syndrome is a rare autosomal recessive disorder caused by pathogenic variants in the *BLM* gene and is characterized by early-onset malignancy, growth deficiency, metabolic abnormalities, and cutaneous findings. We report the case of a 33-year-old woman with bilateral early-onset breast cancer, lipodystrophy, severe hypertriglyceridemia complicated by pancreatitis, and type 2 diabetes mellitus. Initial targeted genetic testing did not identify a causative variant. Comprehensive multigene testing subsequently revealed a homozygous likely pathogenic splice-site variant in *BLM* (c.98+1G>T), establishing the diagnosis of Bloom syndrome. This case expands the clinical and genetic spectrum of Bloom syndrome and illustrates that targeted cancer gene panels can fail to detect syndromic cancer predisposition disorders, leading to diagnostic delay. Expanded genetic testing should be considered early in patients with multiple primary malignancies accompanied by suggestive syndromic features, as timely diagnosis enables syndrome-adapted management, tumor surveillance, and family counseling.

## Introduction

Bloom syndrome is a rare autosomal recessive disorder caused by pathogenic variants in the *BLM* gene, encoding a RecQ DNA helicase, which is essential for DNA replication, repair, and genomic stability. Loss of BLM function results in chromosomal instability and a profound increase in susceptibility to early-onset malignancies [1]. Fewer than 300 cases have been reported in the Bloom Syndrome Registry since its first description in 1954, and the syndrome is more frequently described in the Ashkenazi Jewish population [2,3]. Heterozygous carriers are typically asymptomatic, although their cancer risk remains unclear [1,4].

Clinically, Bloom syndrome is characterized by growth deficiency that persists throughout life. Patients often present with distinctive craniofacial features (dolichocephaly, narrow face, mandibular and zygomatic hypoplasia) and cutaneous manifestations, including telangiectatic erythema, café-au-lait macules, and hypo- or hyperpigmented skin lesions [1,4]. Body fat mass is generally reduced, sometimes with a distribution resembling lipodystrophy [5]. Endocrine and metabolic abnormalities, including hypothyroidism in approximately 5% of patients, type 2 diabetes mellitus despite low BMI, and secondary dyslipidemia, are well-recognized [1,4]. Immunodeficiency (with hypogammaglobulinemia) predisposes patients to recurrent infections, while reproductive abnormalities, such as male oligo- or azoospermia and female premature menopause, occur frequently [1,4]. The multisystem involvement, combined with the elevated cancer risk, poses significant diagnostic and therapeutic challenges, particularly when the clinical presentation is incomplete or atypical.

We report the case of a young woman with Bloom syndrome, whose clinical course exemplifies the characteristic multisystem involvement, including endocrine dysfunction, dyslipidemia, and bilateral breast malignancy, thereby highlighting the diagnostic and therapeutic implications of delayed recognition of a syndromic cancer syndrome. This case is of particular interest due to the rare occurrence of bilateral breast cancer in Bloom syndrome and the identification of an uncommon pathogenic *BLM* variant. 

## Case presentation

A 33-year-old woman of North African origin was evaluated for a multisystem clinical presentation. She was diagnosed with hypothyroidism at age 15, for which she receives levothyroxine substitution. At age 24, she developed acute pancreatitis secondary to severe hypertriglyceridemia, classified as mild according to the Ranson criteria score. Additional metabolic comorbidities included hepatic steatosis and hypercholesterolemia, as summarized in Table 1. At age 29, she was diagnosed with type 2 diabetes mellitus accompanied by pronounced insulin resistance, requiring high-dose insulin therapy (total daily dose of 72IU). The diabetes-related autoantibodies were negative. Her family history includes three brothers, one of whom has type 1 diabetes mellitus. Her grandparents were consanguineous relatives.

**Table 1:** Laboratory findings in the patient at age 30 and peak lipid levels at pancreatitis diagnosis HbA1c: glycated hemoglobin; CRP: C-reactive protein; ALT: alanine transaminase; AST: aspartate transferase; ALP: alkaline phosphatase; LDL: low-density lipoprotein; TSH: thyroid-stimulating hormone

Laboratory Parameter	09/2022 (age 30)	Reference
HbA1c (%)	7.5	4-6%
HbA1c (IFCC, mmol/mol)	58	<42
C-peptide (nmol/L)	1.98	0.37-1.47
Glycemia (mg/dL)	224	70-100
CRP (mg/dL)	1.7	<5
ALT (UI/L)	64	<33
AST (UI/L)	38	<32
ALP (UI/L)	64	35-104
Gamma GT (UI/L)	143	jun/42
Lipase (U/L)	(pancreatitis diagnosis: 1 255	23-300
Total cholesterol (mg/dL)	222 (pancreatitis diagnosis: 1 360)	<190
LDL cholesterol (mg/dL)	108	<100
Triglycerides (mg/dL)	364 (pancreatitis diagnosis: 12 356)	<175
TSH (mU/L)	2.66	0.27-4.2
Anti-TPO (kUI/L)	17	<34
CA 15-3 (kU/L)	27	<26

On physical examination, her weight was 53 kg, and her height was 149 cm (BMI 23.9, Z score -1.43). She exhibited a narrow face and mild mandibular hypoplasia. Dorsocervical fat accumulation was noted. Cutaneous examination revealed areas of hyper- and hypopigmentation, as well as a café-au-lait spot on the right elbow. She did not exhibit photosensitivity or telangiectasia, and she had no history of recurrent infections. There was no history of developmental delay or intellectual disability.

At age 27, she was diagnosed with triple-negative breast carcinoma of the right breast (Ki-67 40%, stage T2N0M0), treated with (neo)adjuvant chemotherapy (Epirubicin, Cyclophosphamide, Paclitaxel, and Carboplatin), followed by lumpectomy and adjuvant radiotherapy. Treatment-related side effects included dermatitis and significant hematological toxicity. Genetic analysis in 2019 did not identify pathogenic variants in the following genes: *BRCA1, BRCA2, PALB2, TP53, CHEK2, ATM, RAD51C, RAD51D, BRIP1, MLH1, MSH2*, and *MSH6*; notably, *BLM* was not included in this panel.

**Figure 1:**
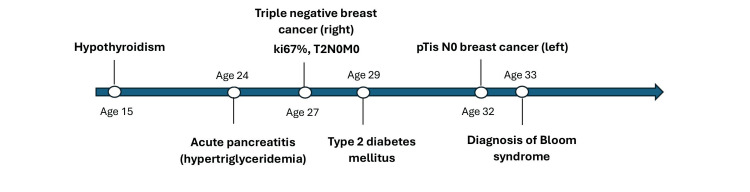
Clinical timeline of endocrine, metabolic, oncologic, and genetic events

At age 32, she developed a second primary breast cancer in the left breast (pTis N0), estrogen receptor (ER)-positive, progesterone receptor (PR)-negative, human epidermal growth factor receptor 2 (HER2)-negative, treated with lumpectomy, radiotherapy, and adjuvant tamoxifen. Despite prior negative genetic testing, the combination of early-onset bilateral breast cancer, lipodystrophy, metabolic abnormalities (diabetes and severe hypertriglyceridemia with pancreatitis), and consanguinity raised suspicion of an underlying syndromic etiology. Comprehensive genetic analysis was performed (exome sequencing), including gene panels for insulinopathies (83 genes), lipodystrophy syndromes (34 genes), and hereditary breast/ovarian cancer. This analysis identified a homozygous likely pathogenic (class IV) variant in the *BLM* gene (c.98+1G>T) (Table 2). mRNA analysis demonstrated that this variant disrupts normal splicing, leading to the loss of exons 1 and 2, thereby abolishing normal BLM protein function. Her parents were not available for segregation analysis. Figure 1 shows the clinical timeline of the patient's endocrine and oncologic events and the diagnosis of Bloom syndrome.

**Table 2:** Genetic analysis identifying a homozygous likely pathogenic BLM splice-site variant (c.98+1G>T) Chr: chromosome, HOM: homozygous

Gene	Transcript	Chr	Position	Exon	Coding	Status	Evaluation
BLM	NM_000057	15	90747491	2	c.98+1G>T	HOM	4-probably pathogenic

## Discussion

We report the case of a young woman of North African origin with a confirmed diagnosis of Bloom syndrome presenting with bilateral early-onset breast cancer, severe hypertriglyceridemia complicated by acute pancreatitis, type 2 diabetes mellitus, short stature, lipodystrophy-like fat distribution, and characteristic cutaneous findings. In retrospect, her clinical presentation closely matched the established phenotype of Bloom syndrome. Despite early-onset breast cancer, the diagnosis remained unrecognized until subsequent contralateral breast cancer, when comprehensive multigene testing was performed.

Patients with Bloom syndrome are susceptible to a wide spectrum of malignancies [1,4]. Among the 290 patients in the Bloom Syndrome Registry, 53% (155 patients) developed cancer. Breast cancer was reported in 29 of 290 registered individuals, while 35% of individuals in the Bloom Syndrome Registry developed multiple malignancies [6]. To our knowledge, based on the available published literature, this appears to be the first reported case of bilateral breast cancer in a patient with Bloom syndrome. This markedly elevated cancer risk warrants early tumor screening starting in childhood, together with annual metabolic surveillance [4].

The c.98+1G>T variant identified in this patient is rare. It has been reported in only 9 heterozygous individuals in the Genome Aggregation Database (gnomADv4.1.1) and was previously described only once in a patient with Bloom syndrome [7,8]. mRNA analysis confirmed that this variant disrupts normal splicing, resulting in the loss of exons 1 and 2 and thereby abolishing normal BLM protein function [9]. The patient's homozygous state, likely facilitated by the known consanguinity of her grandparents, is consistent with the autosomal recessive inheritance pattern of Bloom syndrome [4]. 

An important observation in this case is that targeted genetic testing in 2019 did not identify the underlying syndromic diagnosis. The hereditary breast cancer panel tested 12 established susceptibility genes, but did not include *BLM*. This highlights a limitation of targeted cancer gene panels: syndromic causes can remain undetected when genetic testing is restricted to selected genes and the broader phenotype is not yet recognized. In this patient, Bloom syndrome was diagnosed only after a comprehensive multigene panel integrating panels for insulinopathies, lipodystrophy, and hereditary breast cancer was performed. This underscores the importance of maintaining a broad differential diagnosis in young patients presenting with multiple primary cancers, particularly when accompanied by additional syndromic features and consanguinity.

From a therapeutic perspective, this case raises important questions regarding treatment tolerance in Bloom syndrome. Bloom syndrome patients are generally assumed to exhibit heightened sensitivity to genotoxic therapies. Current treatment recommendations are therefore based solely on expert opinion, case reports, and small case series. Clinical practice guidelines suggest initiating chemotherapy at 50% of the standard weight-based dose, with particular caution regarding alkylating agents [1,4,10]. This patient received full-dose chemotherapy for her first breast cancer, as the diagnosis of Bloom syndrome had not yet been established, and she experienced significant hematological toxicity during therapy. Although hematological toxicity is an expected side effect of this regimen, its occurrence is relevant in retrospect, given the concern for heightened sensitivity to genotoxic agents in Bloom syndrome [1].

Additionally, the optimal radiotherapy dose in Bloom syndrome remains uncertain. While clinical practice guidelines recommend dose reduction based on theoretical radiosensitivity, a published case series has reported good tolerance of standard-dose radiotherapy in individual patients [1,10]. This underscores the importance of timely diagnosis, as knowledge of an underlying DNA repair deficiency may directly inform therapeutic decisions and limit unnecessary genotoxic exposure. Given the rarity of Bloom syndrome, no prospective trials exist to our knowledge.

Following the diagnosis of Bloom syndrome, the patient requires intensified and structured cancer surveillance according to established recommendations [4]. These include regular screening for colorectal cancer, lymphoma, leukemia, and breast cancer, as well as continued metabolic monitoring [4]. Furthermore, given the autosomal recessive inheritance of Bloom syndrome and the family's known consanguinity, genetic counseling and cascade testing of family members, including her brothers, are strongly recommended to identify carriers and assess reproductive risks [4].

## Conclusions

This case underscores the importance of considering Bloom syndrome in patients presenting with early-onset malignancy combined with metabolic abnormalities, growth restriction, and cutaneous features, particularly in the context of consanguinity. Although based on a single case, our findings suggest that expanded multigene testing may be considered in selected patients with such presentations, as targeted gene panels can miss syndromic diagnoses. Timely diagnosis has important implications, as it carries important implications for syndrome-informed treatment, intensified tumor surveillance, and family counseling. Optimal care requires coordinated multidisciplinary management given the combination of metabolic and oncologic comorbidities.
